# Development of a Novel Robust Approach for Unveiling the Stretchiness of Cheese

**DOI:** 10.1111/jtxs.70012

**Published:** 2025-03-04

**Authors:** Pietro Andrigo, Sara Spilimbergo, Belinda P. C. Dewi

**Affiliations:** ^1^ Department of Industrial Engineering University of Padova Padova Italy; ^2^ Fermify GmbH Vienna Austria

**Keywords:** cheese, meltability, mozzarella, stretchability, texture analyzer

## Abstract

This study proposed a method for objectively measuring the stretchiness of cheese using a texture analyzer equipped with a double‐sided fork probe. The method measures the force required to stretch melted cheese samples at a constant speed. Using a central composite design (CCD) experimental approach, the impact of test parameters (i.e., sample quantity, temperature, speed) has been evaluated. Subsequently, an optimal combination of the test parameters (i.e., 66°C, 17.5 mm/s, 6.7 g sample) has been proposed. These aimed to minimize variability and ensure the reproducibility of the results in the stretchiness of the observed cheese. The key stretch descriptors' significance to describe the stretchiness of cheese, namely peak force, force and work at a distance of 40 mm, and breaking distance has also been indicated. With the proposed method, changes in the stretch properties of Mozzarella during storage at 4°C were able to be monitored. An initial decrease was observed up to day 7, followed by a slight increase by day 25, suggesting changes in the cheese protein network. The method was then used to compare the properties of 15 dairy‐based cheeses and 6 vegan Mozzarella‐style cheese alternatives. The peak force and filament strength (i.e., force at a distance of 40 mm) were used to cluster various cheeses showing distinct stretch profiles. Mozzarella cheeses generally showed lower resistance to filament formation compared to other dairy‐based cheeses, while vegan cheeses exhibited minimal to no filament formation and lower meltability. By comparing and clustering the stretchiness properties of dairy cheeses and their vegan alternatives, the present method can be used to guide researchers and product developers on better profiling the stretch properties of target cheese and subsequently producing (new) products with a more desirable stretch profile.

## Graphical abstract

This study presents a texture analysis method to measure cheese stretchiness, evaluating test parameters to optimize reproducibility. The method was used to profile the stretch properties of dairy and vegan Mozzarella‐style cheeses, providing insights to guide product development.

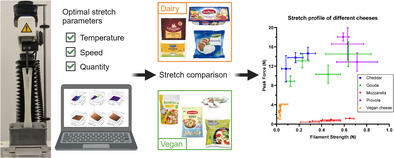

## 1 Introduction

Mozzarella cheese is among the most popular cheeses in diverse culinary traditions. As part of the ‘pasta filata’ cheeses, its production process is characterized by stretching the curd in hot water, resulting in a distinct smooth surface texture. The functional properties of mozzarella (e.g., melting, stretching) contribute significantly to its applications, as well as to consumer preference and acceptance (Smith et al. 2018; Ah and Tagalpallewar 2017). For example, its meltability and stretchability are critical in its applications as pizza toppings. Here, meltability refers to the cheese's ability to soften and flow smoothly when heated, while stretchability (known also as stringiness or stretchiness) describes the ability of melted cheeses to form elongated and continuous strands when pulled (Ah and Tagalpallewar 2017). These properties are influenced by the interaction of the cheese's main components, such as water, caseins, whey, and fat (McMahon et al. 2005).

In recent years, consumer demand for vegan and plant‐based cheeses has been escalating. The global market for vegan cheese substitutes is projected to expand at a compound annual growth rate of 10.64% from 2024 to 2030 (Research and Markets 2024). Despite the increasing demand, developing vegan cheese analogs that meet consumers' preferences is extremely challenging. One of the most difficult properties to imitate is stretchiness (Grossmann and McClements 2021). The relevance of the stretchiness attribute is crucial but subjective, as unstandardized methods are employed for the measurements. The most common cheese stretch assessment relies on the fork test. This approach involves raising a fork through the melted cheese until the strands break. The breaking distance is referred to as “stretchiness”. While valuable for initial evaluations due to its simplicity and quickness, the fork test used in previous studies may lack reproducibility and consistency concerning the way it usually performs, which is highly influenced by the person conducting the test. Factors such as fluctuations in temperature, subjective measurement of the breaking distance, lack of control over stretching speed, and inconsistencies in sample handling and preparation contribute to significant variability in the results (Guinee and O'Callaghan 1997; Lim and Cheng 2024). Thus, the need for a more objective measurement technique is desired.

**FIGURE 1:**
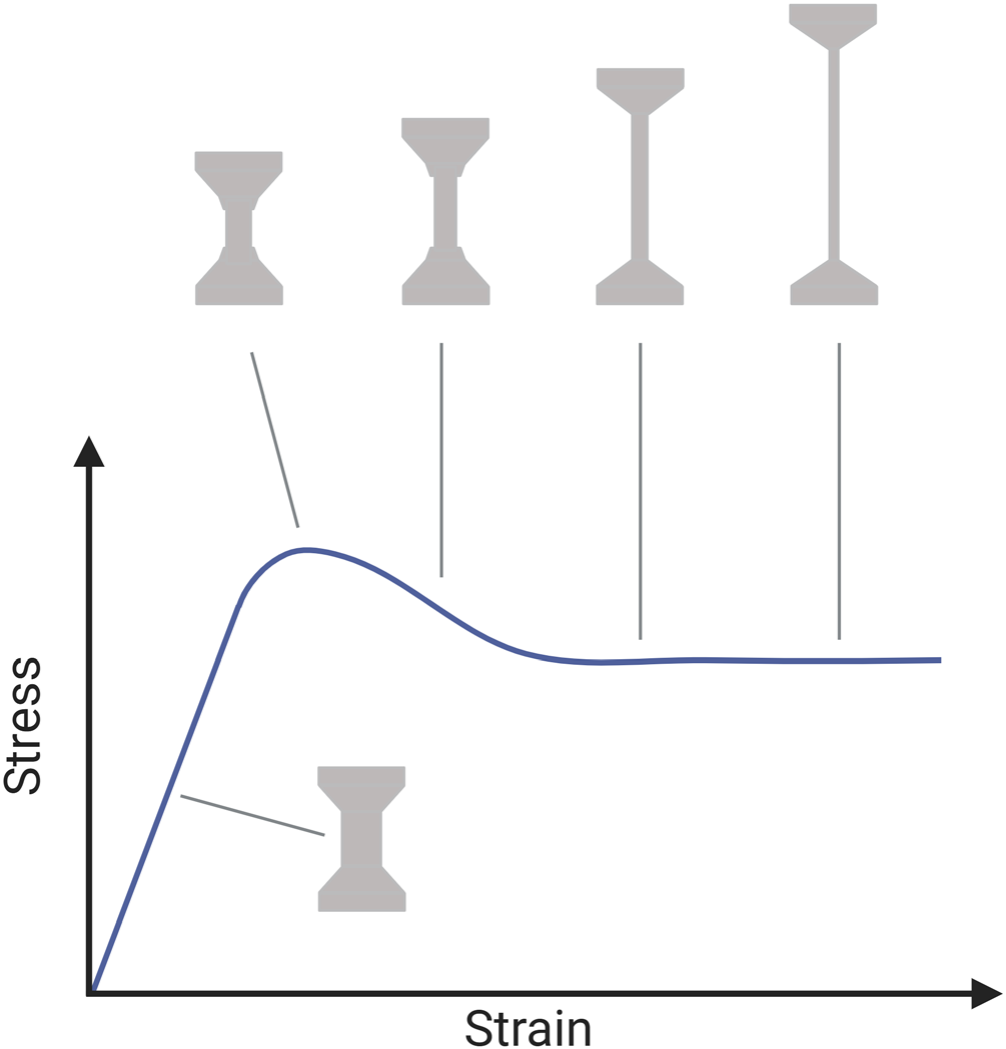
A typical stress–strain curve resulting from the tensile test of some plastic polymers.

Various attempts have been made to objectively measure the stretchiness of cheeses since the early 1990s. Most of them rely on the utilization of a texture analyzer to apply a pull force at a constant speed while measuring the responding force generated (Fife et al. 2002; Lim and Cheng 2024; Ma et al. 2012; Salunke et al. 2022). This approach allows the collection of parameters that can be further translated into the stretch properties of the sample. As noted by Ma et al. (2012), the tensile behavior of melted cheese resembles that of some plastic polymers, where similar standard tests are used (Draghi 2017; Ma et al. 2012). Like cheese, during the tensile test, the pull results in a local decrease of the cross‐sectional area with a consequent stabilization and propagation called necking, where a relatively large amount of strain is localized disproportionately (*confer* (cf.) Figure 1). While the method for tensile testing of polymers has been well established, no standard procedure for cheese has been implemented in industry or academic research. A significant improvement came with the introduction of the cheese extensibility rig developed by Stable Micro Systems (Lim and Cheng 2024). This probe is an enhanced version of the fork test, where a double‐sided fork is placed in the bottom of a pot and covered with an amount of cheese. The cheese is then melted over the fork, a retaining insert is placed to hold the sample tight to the pot base, and then the fork is lifted by a texture analyzer at a constant speed. During the pull, filaments are formed, and the responding force is measured. This probe has been used in previous studies (Dai et al. 2018; Feng et al. 2021; Guinee et al. 2015; Gulzar et al. 2020; Salunke et al. 2022). However, a direct comparison of the data among articles is intricate because of the different protocols and responding parameters considered. Thus, this drives the need for a standardized method.

This present study aimed to propose an objective way to assess the stretchiness of dairy‐based cheeses or their alternatives (e.g., vegan cheese) by using a texture analyzer. It is hypothesized that, as with other empirical tests using a texture analyzer, the responding force is influenced by specific test conditions. The results are therefore a snapshot of the mechanical properties of the sample under specific test parameters, including speed, temperature, sample quantity, and probe geometry. For instance, at a lower test speed, the sample would have more time to flow, and therefore the force measured would be lower (Guinee and O'Callaghan 1997; Kazemeini and Rosenthal 2022; Peleg 2019). Thus, in the current study, the impact of test conditions on the test results was also considered. Furthermore, it was also investigated whether the proposed method would only work for mozzarella or any other stretchy cheeses, such as Gouda or Cheddar. The effect of the shelf life on the stretchiness of mozzarella was also considered by recording stretch properties during a storage period of 25 days. The latter also exhibits diverse applications of the proposed method.

## 2 Materials and Methods

### 2.1 Materials

Fifteen commercially available dairy‐based cheeses and six commercially available vegan mozzarella‐style cheese substitutes were assessed in this study. For the dairy‐based cheeses, it included bovine mozzarella, bovine Gouda, mild bovine Cheddar, and bovine Provola. All cheeses were purchased from local markets (Spar and Billa, Vienna, Austria), stored at 4°C, and utilized within 48 h. The complete list of the samples, as well as their detailed compositions is depicted in Table 1. Here, the compositions of the cheeses were obtained from the product label provided by the manufacturer of the relevant cheese.

**TABLE 1:** Composition and texture properties of different cheeses. Values are presented as mean ± standard deviation (s.d.). Lowercase letters (a, b, c, etc.) denote cheeses with statistically similar values of a given property, with each property represented as a column (α =0.05).

Cheese	Composition (g/100 g)							pH (*n* = 3)	Stretch properties				Meltability (%)
	Fat	Saturated fat	Carbohydrates	Sugars	Proteins	Salt	Calculated moisture		Peak force (N)	Filament strength (N)	Work at 40 mm (mJ)	Breaking point (mm)	
**Bovine Mozzarella**													
Clever Mozzarella Light (Clever, Austria)	8.5	5.5	1.0	1.0	20.0	0.5	70.0	5.91 ± 0.10	0.88 ± 0.14	0.484 ± 0.077	25.41 ± 3.90	> 215	229 ± 41^d^
Denree bio‐Mozzarella (Dennree, Germany)	19.0	12.0	1.0	1.0	17.0	0.6	62.4	6.10 ± 0.04	0.41 ± 0.06	0.296 ± 0.084	13.50 ± 2.36	207.5 ± 13.9	344 ± 100^b^
Galbani Mozzarella cucina (Galbani, Italy)	19.0	13.0	2.2	1.6	20.0	0.9	57.9	5.90 ± 0.02	1.17 ± 0.12	0.649 ± 0.038	37.56 ± 3.71	> 215	253 ± 21^cd^
Galbani Mozzarella (Galbani, Italy)	18.0	13.0	2.0	1.0	17.0	0.7	62.3	6.16 ± 0.01	0.62 ± 0.10	0.397 ± 0.081	20.43 ± 3.09	153.8 ± 15.5	342 ± 46^b^
Züger Bio (Züger, Switzerland)	19.0	12.0	1.0	1.0	19.0	0.6	60.4	5.81 ± 0.02	0.71 ± 0.08	0.507 ± 0.063	23.65 ± 2.50	164.7 ± 31.9	350 ± 49^ab^
**Vegan cheese**													
Bedda for pizza (Ethiconomy Services, Germany)	24.0	21.0	22.0	0.5	1.8	2.0	50.2	3.81 ± 0.06	3.89 ± 0.27	0.032 ± 0.010	20.59 ± 0.96	*	5 ± 5^g^
Mondarella, gratinello (Mondarella, Germany)	24.5	1.8	22.7	19.1	1.6	1.2	50.0	4.49 ± 0.19	4.14 ± 0.14	0.074 ± 0.027	25.65 ± 0.65	39.9 ± 1.7	13 ± 12^fg^
Mozzarisella Klassich (Frescolat, Italy)	16.0	14.0	8.5	0.3	0.7	1.5	73.3	3.76 ± 0.03	3.00 ± 0.46	0.033 ± 0.005	12.14 ± 0.92	*	69 ± 15^ef^
Spar, veganer bio‐Italian style (Spar, Nederlands)	16.0	14.0	8.5	0.5	0.7	1.5	73.3	3.92 ± 0.05	1.77 ± 0.76	0.031 ± 0.003	8.70 ± 2.19	*	2 ± 7^g^
Züger, bio MozzaVella (Züger, Switzerland)	16.0	6.2	2.4	0.0	3.5	1.0	77.1	4.85 ± 0.06	1.27 ± 0.07	0.013 ± 0.003	5.69 ± 0.66	*	23 ± 14^fg^
Violife, mozzarella Geschmack (Violife, Greece)	24.0	22.0	21.0	0.0	0.0	2.2	52.8	4.36 ± 0.12	2.58 ± 0.29	0.023 ± 0.006	14.39 ± 1.79	*	70 ± 26^efg^
**Cheddar**													
Cathedral city, English Cheddar mild (Saputo Dairy, UK)	34.9	21.7	0.1	0.1	25.4	1.8	37.8	4.99 ± 0.04	11.46 ± 2.74	0.084 ± 0.040	49.56 ± 11.00	43.5 ± 10.4	401a ± 49^ab^
Cheddar mild (Original Cheddar Cheese, UK)	35.0	22.0	0.5	0.5	25.0	1.8	37.7	5.24 ± 0.03	13.78 ± 1.06	0.168 ± 0.068	69.84 ± 10.33	146.2 ± 57.6	314 ± 24^bcd^
SPAR, Würziger Cheddar 4 months (Spar, Nederlands)	35.0	22.0	0.5	0.5	25.0	1.7	37.8	5.25 ± 0.05	14.63 ± 1.31	0.278 ± 0.067	86.19 ± 11.49	171.2 ± 24.3	432 ± 59^a^
**Gouda**													
Woerle mid fein (WOERLE, Austria)	26.0	27.0	0.0	0.0	26.0	1.3	46.7	5.39 ± 0.05	8.88 ± 1.10	0.124 ± 0.036	48.99 ± 8.12	> 215	226 ± 37^d^
Schärdinger österreichischer Gouda (Schärdinger, Austria)	26.0	17.0	0.0	0.0	26.0	1.3	46.7	5.60 ± 0.05	14.52 ± 2.59	0.637 ± 0.202	94.71 ± 22.69	> 215	93 ± 20^e^
S‐BUDGET Österreichischer Gouda (Spar, Nederlands)	25.0	17.0	0.0	0.0	24.0	1.1	49.9	5.34 ± 0.07	10.32 ± 1.87	0.457 ± 0.110	64.91 ± 12.80	> 215	205 ± 31^d^
SPAR Natur*pur Bio‐Gouda (Spar, Nederlands)	29.0	22.0	0.0	0.0	24.0	1.2	45.8	5.52 ± 0.05	13.10 ± 1.43	0.229 ± 0.059	65.27 ± 7.69	> 215	381 ± 74^ab^
**Provola**													
Italiamo, Provoletta (Lidl, Germany)	19.0	12.0	0.9	0.9	24.0	1.4	54.7	5.02 ± 0.09	12.87 ± 1.92	0.715 ± 0.183	98.36 ± 17.52	> 215	316 ± 38^bc^
Cucina nobile, Natur provolone (ALDI, Germany)	26.0	17.0	0.7	0.7	23.0	1.8	48.5	5.36 ± 0.09	18.06 ± 0.84	0.591 ± 0.052	122.77 ± 9.73	> 215	301 ± 40^bcd^
Cucina nobile, Provolone Valpadano (ALDI, Germany)	26.0	18.0	0.5	0.5	25.0	1.7	46.8	5.39 ± 0.05	16.60 ± 3.37	0.631 ± 0.143	102.36 ± 26.01	> 215	298 ± 37^bcd^

### 2.2 Methods

#### 2.2.1 Stretchiness Measurement Setup

**FIGURE 2:**
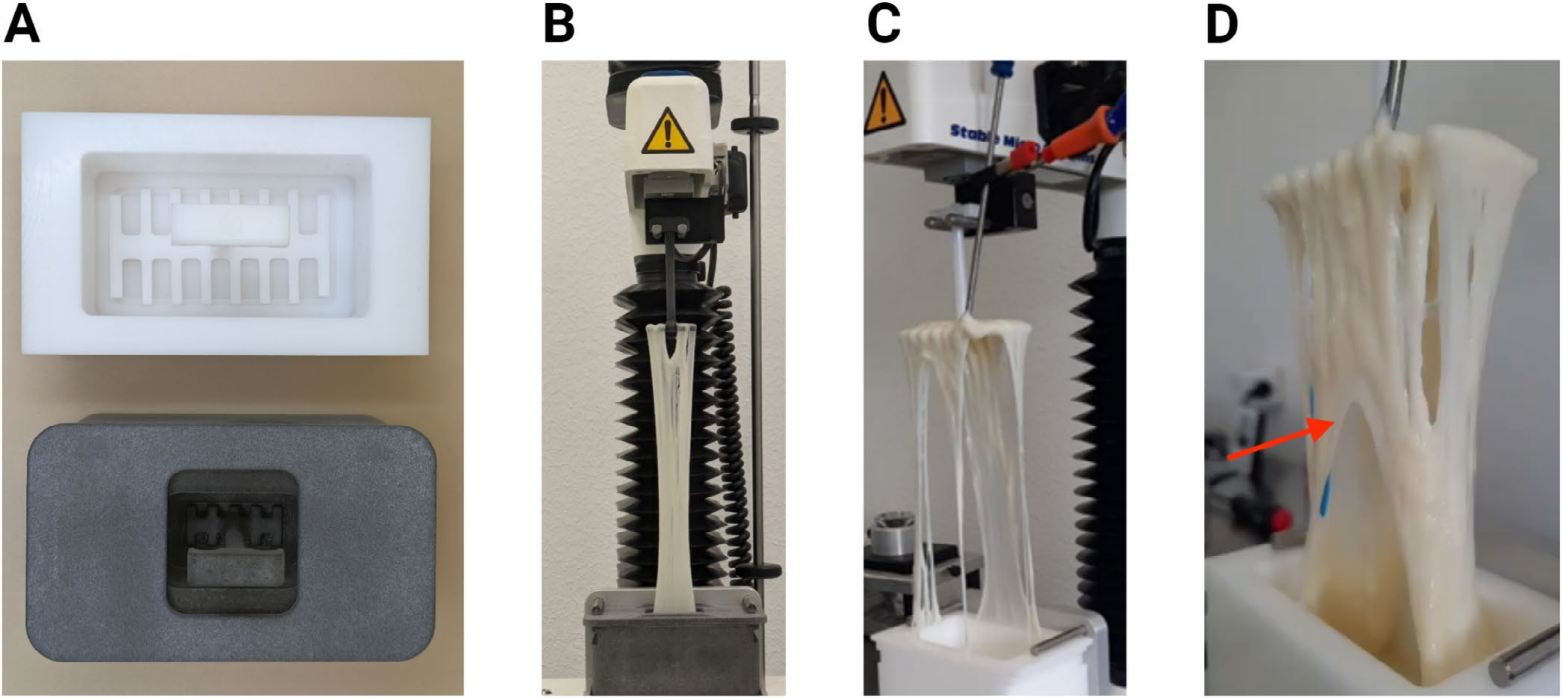
Comparison of cheese extensibility probes. (A) Top view of the Stable Micro Systems cheese extensibility rig (above) and the scaled‐down 3D printed probe used in this study (below). (B) Texture analyzer equipped with the scaled‐down probe during a stretch test of mozzarella sample. (C) Similar setup as in (B), but with the Stable Micro Systems cheese extensibility rig. (D) Detailed view of a slippage problem encountered with some of the Mozzarella samples using the cheese extensibility rig, with red arrows indicating the visible arc in the sample caused by the slippage.

The stretchiness of cheese samples was determined by measuring the responding force during the uniaxial extension of the molten cheese mass at a constant speed using a texture analyzer (TA.XTplus, Stable Micro Systems Ltd., United Kingdom) equipped with a 50 N load cell. During preliminary tests, the Cheese Extensibility Rig probe (i.e., provided by Stable Micro Systems Ltd., United Kingdom) was used. To address the slippage issue observed during preliminary tests, a scaled‐down version of the probe was introduced (cf. Figure 2). This revised probe, 3D printed in Nylon PA12 (Weerg, Italy), has smaller dimensions compared to the original probe provided by Stable Micro Systems. The current dimensions of the fork base are 29 mm × 12 mm, and the internal pot base is 36.5 mm × 18.5 mm. This probe enables the analysis of cheese samples with a maximum weight of 10 g.

The experimental procedure used is similar to that described in the manual of the Stable Micro Systems extensibility probe. It begins with placing the double‐sided fork at the bottom of the pot. The sample is weighed, shredded, and placed inside the pot. The sample had to be equally distributed and cover the entire fork. Subsequently, the pot, along with the fork and sample, is placed for 15 min in a static oven at a temperature of 110°C. This allows the cheese to melt uniformly and to reach a temperature of about 80°C. The probe containing the sample is then removed from the oven and cooled down at room temperature (20°C) until the test temperature is reached (i.e., stretch temperature). During this step, the temperature of the cheese is monitored using a PT100 probe (SCANTEMP PRO 450 equipped with a PT100 1.5 mm probe). Once the stretch temperature is reached, a retaining insert is placed to hold the cheese perimeter tight to the pot base. The assembly of the probe and sample is then securely fastened to the texture analyzer. The tensile test starts within 15 s from when the desired temperature is reached. The fork is then lifted at a constant speed, and the responding force is measured during the extension of 215 mm (cf. Figure 2B).

From the force‐distance curve, four different stretch descriptors can be derived, namely: (1) the peak force (N), which represents the maximum force exerted, usually measured in the first millimeters of extension, (2) the force measured at a distance of 40 mm extension (N), (3) the area under the curve until a distance of 40 mm (mJ), which provides insight into the energy required for the stretch, and (4) the breaking point (mm), indicating the distance at which the cheese sample can no longer withstand the stretching force.

#### 2.2.2 Optimization of Stretch Test Parameters

Test parameters such as sample quantity, test speed, and test temperature can directly affect measurements in the stretch test of cheeses (Fife et al. 2002; Guinee and O'Callaghan 1997). To describe and quantify the impact of these parameters, a response surface methodology has been adopted following a central composite design (CCD). Originally developed by Box and Wilson (1951), CCD is an experimental framework that allows an efficient construction of a quadratic model for the considered response variables (Box and Wilson 1951). The three test parameters were considered: (1) sample size (g), (2) stretch temperature (°C), and (3) test speed (mm/s), while the considered responses (i.e., stretch descriptors) were peak force (N) and filament strength (N). It is noted that filament strength is used throughout the paper and refers to the force at a distance of 40 mm. A total of 15 experimental runs were planned accordingly, as shown in Table 2. Each experimental run was analyzed in four replicates, and the order of the experimental run was randomized to minimize potential bias and account for any uncontrolled variables that may influence the outcomes. The obtained data were modeled to estimate linear, quadratic, and combination effects of test parameters on the responding variables, including peak force (N) and filament strength (N). The optimal values of stretch parameters were based on the flat region of the obtained response surface. A flat region is characterized by a relatively constant response despite small variations in the input parameters. Choosing parameters in a flat region represents the best choice to minimize the response variability caused by the influence of hard‐to‐control factors (Guthrie 2020). An example of a hard‐to‐control factor is the possible uneven displacement of the cheese sample inside the probe. For the optimization, a commercial mozzarella cheese (i.e., Mozzarella cucina from Galbani, Italy) was chosen because of the availability of this specific product in local stores throughout the duration of this study. All the samples were analyzed following the described setup and measured immediately after being removed from the fridge (4°C).

**TABLE 2:** Fifteen experimental runs of a Central Composite Design (CCD) and their responses on peak force and filament strength. The values are presented as mean ± standard deviation (s.d.).

Quantity(g)	Speed (mm/s)	Temperature (°C)	Peak force (N)	Filament strength (N)
6	10	60	1.32 ± 0.20	0.833 ± 0.167
6	10	70	0.53 ± 0.04	0.295 ± 0.014
6	24	60	1.87 ± 0.19	1.223 ± 0.102
6	24	70	0.82 ± 0.04	0.490 ± 0.035
10	10	60	13.15 ± 6.06	0.916 ± 0.148
10	10	70	8.46 ± 0.84	0.345 ± 0.029
10	24	60	21.26 ± 2.61	1.826 ± 0.541
10	24	70	12.27 ± 1.54	0.555 ± 0.068
8	17	60	3.78 ± 1.75	1.083 ± 0.118
8	17	70	4.81 ± 2.25	0.389 ± 0.034
8	10	65	1.48 ± 0.62	0.511 ± 0.064
8	24	65	1.89 ± 0.23	0.869 ± 0.113
6	17	65	1.04 ± 0.18	0.683 ± 0.141
10	17	65	14.79 ± 2.04	0.667 ± 0.100
8	17	65	3.71 ± 2.20	0.651 ± 0.091

#### 2.2.3 Stretch Properties of Mozzarella During the Storage Period

Stretch properties of mozzarella cheese (Mozzarella cucina from Galbani, Italy) were also followed and assessed during a storage period of 25 days at 4°C. All the measurements were conducted at the resulting conditions derived from the optimized method as mentioned in Section 3.2. Five replicates were performed for each time point.

#### 2.2.4 Assessment of Stretch and Melting Properties of Vegan and Dairy Cheeses

For the assessment of the stretch properties, five replicates were performed for each cheese sample following the optimized method. The meltability of the cheeses was simultaneously assessed using a modified Schreiber test (Muthukumarappan et al. 1999). Uniform round pieces weighing 1 g were cut from each sample and placed on a baking tray lined with baking paper. The tray was then heated in a preheated oven at 230°C for 3 min. Photographs of each sample were taken before and after melting. Here, a ruler was placed next to the sample used as a scale. The area of the cheese in the photographs, both before and after heating, was calculated using ImageJ software (version 1.54). Six replicates were conducted for each sample.

## 3 Results and Discussion

### 3.1 Defining the Stretch Descriptors

**FIGURE 3:**
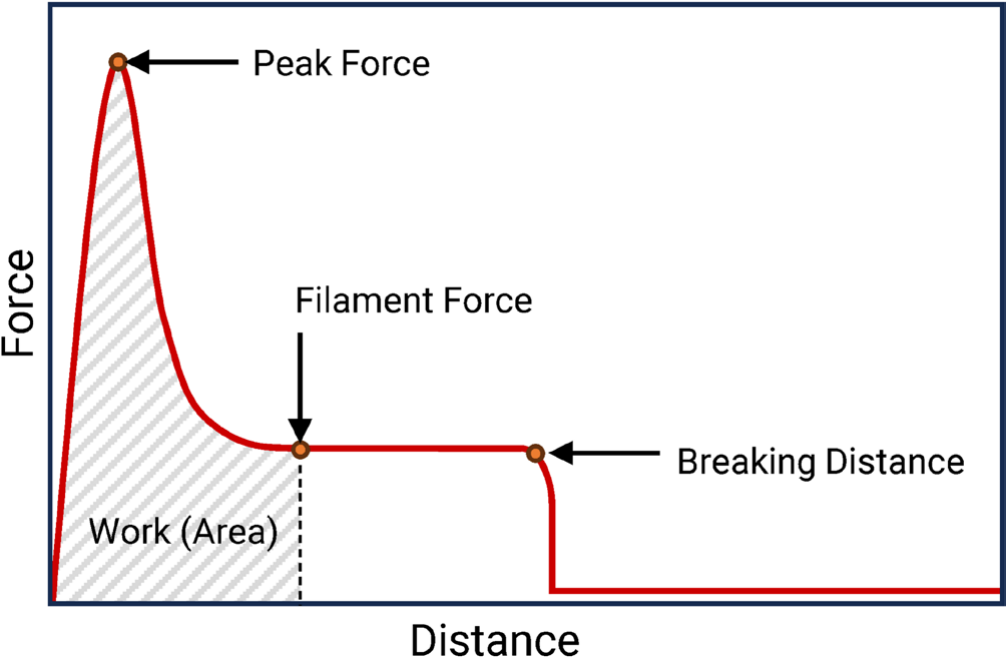
Typical stretch profile of melted cheese and the key stretch descriptors (i.e., peak force, filament strength at a 40 mm stretch, work done during stretching, and breaking distance).

A texture analyzer equipped with a double‐side fork has been utilized to assess the stretch properties of cheese in various previous studies, and different stretch descriptors have been considered from the responding force‐distance curve. Table 3 lists the stretch descriptors that were previously reported in the literature. Four stretch descriptors are considered in this study, highlighted in Figure 3, namely, (1) peak force (N), (2) force at 40 mm (N), (3) total work done to stretch to 40 mm (mJ), and (4) breaking distance (mm). The peak force, usually located after 3–5 mm, represents the initial resistance to moving the fork through the melted cheese mass (Guinee et al. 2015; Lim and Cheng 2024). Once the peak force is overcome, the force measured decreases rapidly, and it stabilizes during the filament elongation. The force at 40 mm of extension was evaluated in this study and referred to as filament strength (N). At 40 mm of elongation, the fork is just entirely out of the pot, allowing for visual observation of the filaments. Measuring this value at a larger distance may introduce errors due to the temperature drop of the filaments occurring during the extension (Ma et al. 2012) and because of the increasing chances of filament breakage with a consequent drop in the responding force measured. However, the temperature drop at 40 mm was considered negligible, as it occurred just 2.29 s after the start of the test at an extension speed of 17.5 mm/s. The breaking distance, which is the distance traveled by the fork before filament breakage occurs, was considered a relevant parameter. However, due to instrumental limitations (specifically, a maximum stretch extension of 215 mm), this point was not always reached, especially for highly stretchable cheeses. Consequently, the breaking distance was evaluated only up to 215 mm. It is further noted that to ensure a visible observation of the filaments, 40 mm was considered the lower limit for the breaking distance.

**TABLE 3:** Previously reported stretch descriptors used to define the stretchiness of cheese.

Stretch descriptors	Reference
Force at 100 mm	Dai et al. 2018
Gradient from 100 to 270 mm	
Ratio between force at 270 mm and peak force	Feng et al. 2021
Work to stretch 100 mm	Grasso et al. 2024
Work to stretch 380 mm	Guinee et al. 2015
Peak force	
Force at 380 mm	
Work to stretch 380 mm	McCarthy et al. 2016
Work to stretch 340 mm	To et al. 2023
Peak force	This study
Force at 40	
Breaking distance	
Work to stretch 40 mm	

Furthermore, in tensile tests, the area under the force‐distance curve can be considered, especially from the beginning of the extension until the failure of the sample. This is also referred to as ‘toughness’ (Draghi 2017). However, in cases where the sample does not break, such as with very stretchy cheese or very ductile materials, the area under the curve is measured up to a specific distance of extension. This allows for the comparison of energy absorption during the stretch test up to a specific distance (To et al. 2023; Grasso et al. 2024). This parameter was evaluated until a fixed distance of 40 mm, referred to as the work to stretch 40 mm (mJ). Table 1 presents the values for the four stretch descriptors derived for all cheeses considered in this study. This aims to provide a complete overview of the stretch properties of the studied cheeses. It is noted that during the optimization phase, only peak force and filament strength were considered as key responses. These two stretch descriptors were able to better describe the shape of the force‐distance graph until 40 mm, therefore the stretch behavior of cheese. It is further noted that in the previous studies, as reported in Table 3, values of force greater than 40 mm were also considered as stretch descriptors. However, in the present study force values measured at this distance (i.e., above 40 mm) were not considered because of the aforementioned limitations, including a drop in temperature and potential breakage of filaments.

### 3.2 Optimization of Stretch Test Parameters

**FIGURE 4:**
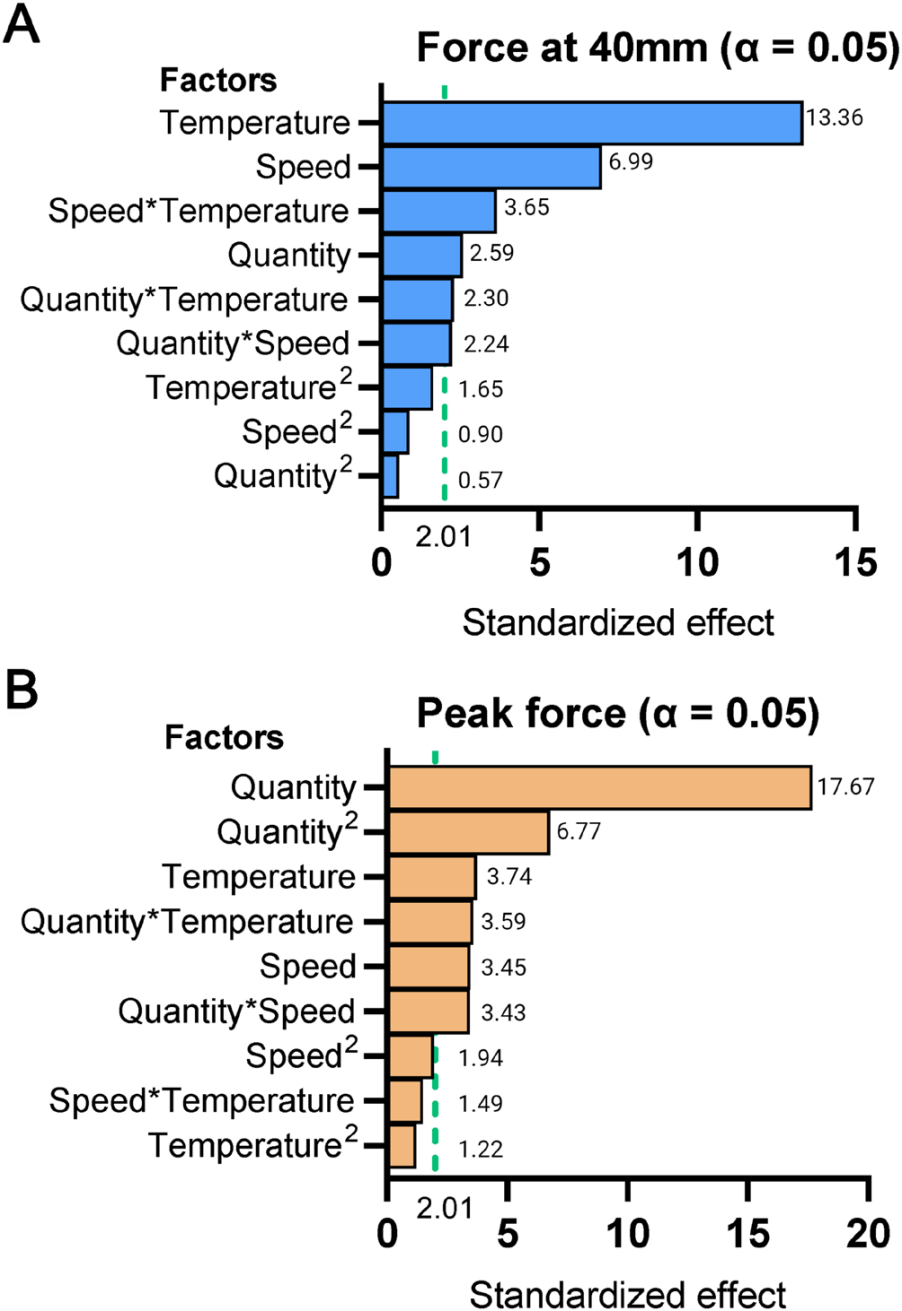
Pareto charts of standardized effects for responses of filament strength and peak force. Standardized effects for filament strength response (A) and peak force response (B). The vertical lines in the charts (i.e., at the value of 2.01) represent the threshold of significance at an alpha level of 0.05, highlighting factors that significantly influence the responses.

The resulting data derived from executing the central composite design (CCD) plan is shown in Table 2. These data were used to compute a polynomial quadratic model for the response variable of peak force (N) and for the filament strength (N) (*R* ^2^ = 0.90). The linear, quadratic, and interaction effects of the three parameters, including sample quantity (g), test speed (mm/s), and temperature (°C), were estimated using Minitab software (Minitab software, Minitab Inc., State College, PA, USA). Standardized effects of these parameters are presented in the form of Pareto charts (cf. Figure 4), which facilitate the comparison of their relative influences. In peak force response, stronger linear and quadratic effects are shown for sample quantity. Lower but still significant linear effects have been estimated for speed and temperature, as well as for the combination effect between quantity and speed and between quantity and temperature. No significant effects are shown for the interaction between speed and temperature and for the quadratic effect of both temperature and speed (*p* > 0.05). In the response of force measured at 40 mm of extension (i.e., filament strength), significant linear effects of the three factors are shown, along with interaction effects between quantity and temperature and between quantity and speed. No significant quadratic effects are present for any of the factors (*p* > 0.05).

**FIGURE 5:**
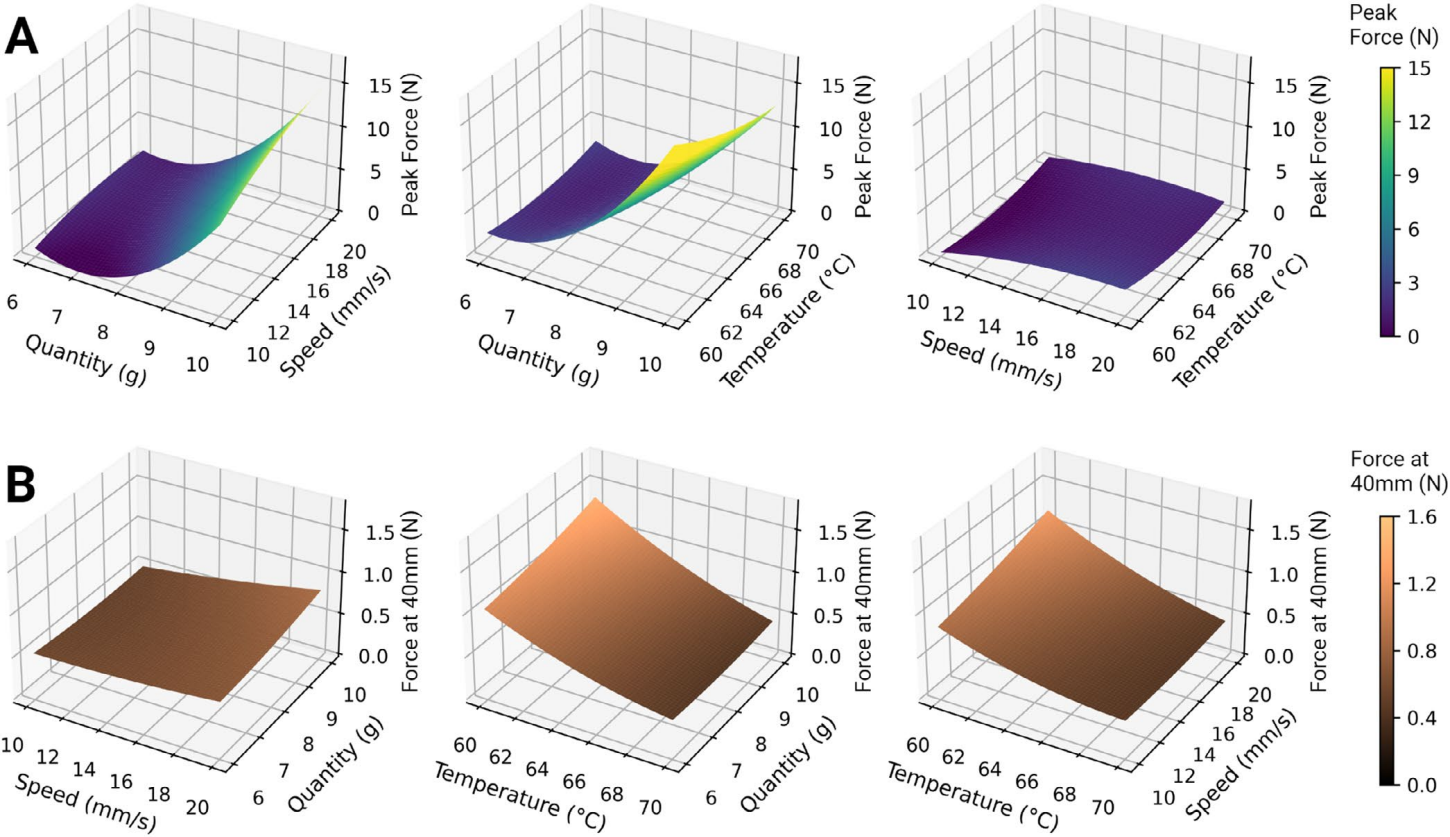
Surface plots for peak force and filament strength responses. Surface plots show the effects of sample quantity, test speed, and test temperature on peak force (A) and filament strength (B), with one factor fixed in each plot (sample quantity = 6.7 g; test temperature = 66°C; test speed = 17.5 mm/s).

Figure 5 presents the response surface plots for both peak force and filament strength. Here, each plot illustrates the interplay between two of the three parameters studied in the CCD while maintaining the third parameter fixed at the optimized value found. It can be observed that the filament strength responses appear predominantly linear, meaning that increasing sample quantity, test speed, or test temperature within the considered range leads to a linear increase or decrease in the response. In contrast, peak force response plots reveal a pronounced quadratic effect for sample quantity. It reaches an exponentially steeper gradient by increasing the sample size from 6 to 10 g. Response peak force in such a steep region can result in larger variability due to small changes in the input factor (i.e., sample quantity). This factor can be influenced by the possible uneven distribution of the sample over the fork. This type of situation can easily occur with complex matrices like cheese, representing a hard‐to‐control factor. Ma et al. (2012) also observed significant variability in the peak force measurement during the tensile test of cheese. Due to this inconsistency, they chose to also consider other responses that were more stable, such as the inflection point after the occurrence of necking and the gradient in the force measured after the peak. In this study, this issue was addressed by selecting test parameters in a flat region of the response surface of peak force. By focusing on the flat point of the response surface, the sensitivity of the peak force to variations in the test parameters can be minimized. Subsequently, it reduces the variability in the results (Guthrie 2020). The flattest point of the surface for peak force was calculated (i.e., the algorithm used is reported in Supporting Information) and rounded, resulting in a stretch temperature of 66°C, stretch speed of 17.5 mm/s, and sample quantity of 6.7 g. These stretch parameters are proposed to be used to measure the stretchiness of cheese, resulting in the least variability of the defined stretch descriptors. It is noted that this optimization was made using a specific probe mentioned in this study. Different probe geometry might lead to different results, as the probe geometry influences the way the cheese is stretched. It is also noted that the results derived from the texture analyzer are snapshots of the mechanical properties of the sample under specific test parameters, including speed, temperature, sample quantity, and probe geometry (Kazemeini and Rosenthal 2022). To our knowledge, no previous studies have attempted to optimize the process parameter for tensile testing aiming for food studies.

Additionally, using the optimized stretch parameters, the changes in mozzarella's stretchiness over time can be followed. This will be useful for applications, for instance, during quality control of cheese, ensuring the consistency of the product throughout its shelf life. Furthermore, the proposed parameters also enabled us to cluster various cheeses based on their stretchiness profile. This can provide valuable insights into product development, for example, better describing the target texture for the new product based on the stretchiness profile. The upcoming Sections 3.3 and 3.4 will explore these two applications in detail.

### 3.3 Stretch Properties of Mozzarella During the Storage Period

**FIGURE 6:**
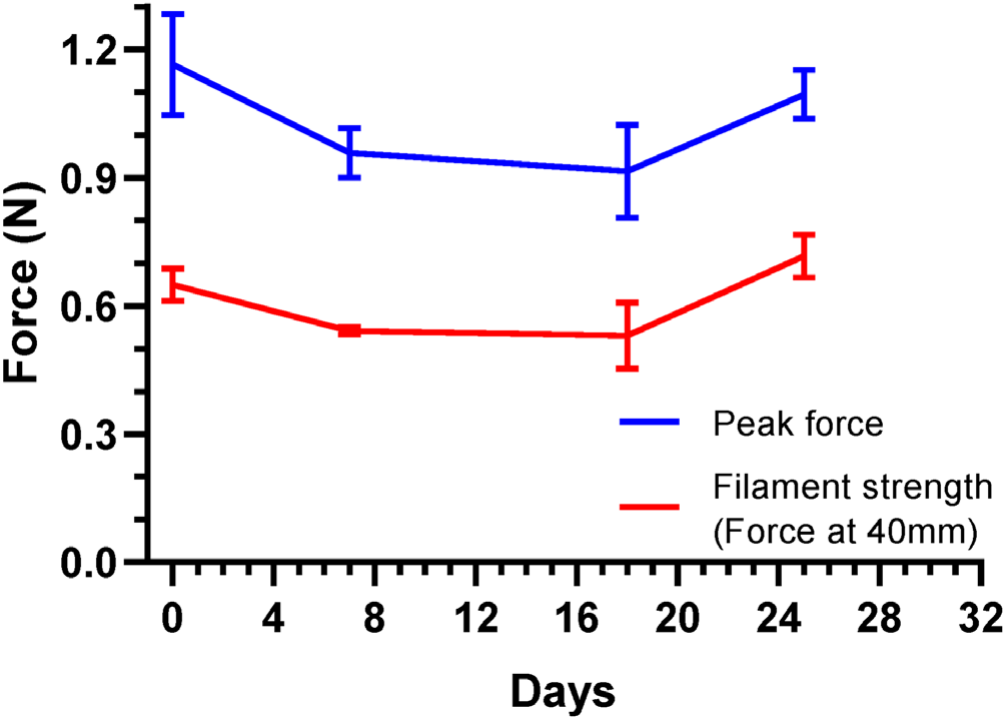
Peak force (N) and filament strength (N) measured during 25 days of storage for a Mozzarella “Galbani Cucina”. Vertical bars represent the standard deviation (s.d.) of the measurements (*n* = 5).

The stretchiness of mozzarella, and therefore its acceptance, undergoes significant changes during the storage period before being consumed. This is primarily due to changes in the calcium balance throughout the storage period, which influence proteolysis and water mobility (Correia Gonçalves and Cardarelli 2021). In the initial weeks of storage, there is a partial dissociation of proteins from the protein matrix. This dissociation leads to a decrease in the stretch properties of the cheese (Smith et al. 2018). Following this phase, the textural properties of the cheese are primarily affected by proteolytic reactions, alterations in water binding capacity, and fat leakage (Ah and Tagalpallewar 2017). Assessing stretchability during the storage period can help to develop stretchy cheeses that have a consistent stretchy profile during the promised shelf life. Using the optimized test parameters mentioned in section 3.2, stretch properties (e.g., filament strength, peak force) were measured for a mozzarella over a storage period of 25 days at 4°C. The results are shown in Figure 6. A significant decrease in both filament strength and peak force was observed between day 0 and day 7 of storage (*p* < 0.05). Similar values were maintained until day 18, while on day 25, an increase in both peak force and filament strength was measured (p < 0.05). From Figure 6 it can be visually noticed that peak force and filament strength show similar trends during the storage period. These exhibited a decline after 7 days, then an increase in the last week of storage. This pattern suggests a potential direct correlation between these two variables in the analyzed cheese sample. This correlation implies that the resistance of the filament to an extension largely contributes to the value of the peak force. This becomes particularly significant when considering that a higher responding force is expected due to the larger cross‐sectional area of the sample at the beginning of the extension before the necking occurs. To our knowledge, there are no previous studies explaining the correlation between the peak force and filament strength. Further studies focusing on these are needed. Moreover, for all the samples measured during the storage test, the breaking distance was observed above the instrumental limit of 215 mm. Thus, a taller texture analyzer arm can be adapted to collect complete information about the breaking distance.

### 3.4 Assessment of Stretch and Melting Properties of Vegan and Dairy Cheeses

**FIGURE 7:**
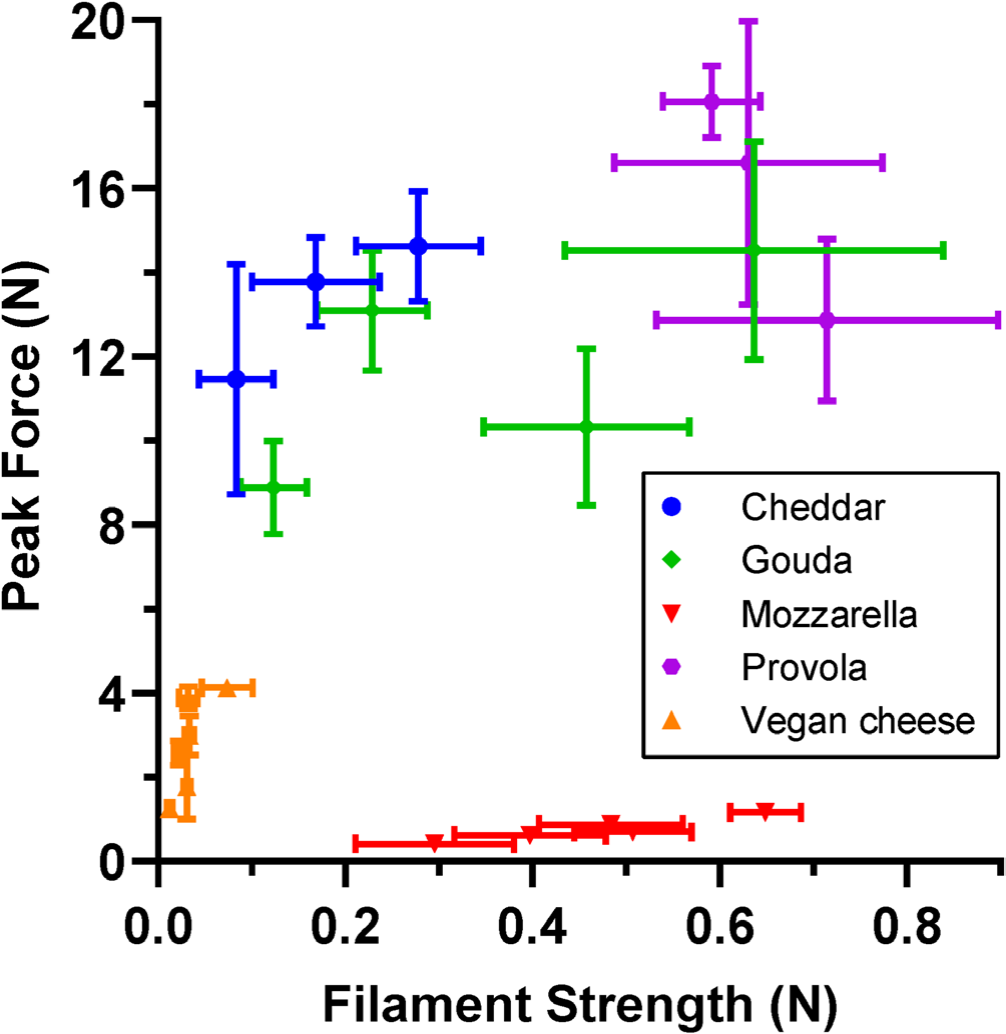
Stretch parameters of 21 different cheeses, as described by peak force and filament strength. Each point on the plot corresponds to one cheese sample, with different colors representing different cheese categories: Gouda, Mozzarella, Vegan, Provola, and Cheddar. Error bars represent the standard deviation (s.d.) for both peak force and filament strength (*n* = 5).

The stretchiness of 21 different cheeses was assessed, including 15 dairy‐based and six vegan mozzarella‐style cheeses. Dairy‐based cheeses were chosen for their well‐known ability to form filaments when melted. To the best of our knowledge, no previous research has examined stretch properties across 21 different cheese samples, both dairy‐based and vegan alternatives. All the results are reported in Table 1, while Figure 7 shows the results in terms of peak force and filament strength. In Figure 7, different types of cheese (i.e., Mozzarella, Cheddar, Gouda, Provola, and Mozzarella‐style vegan alternatives) are highlighted with different colors. The graph suggests a distinct cluster of the cheeses based on their stretch properties. Figure 7 shows that all the mozzarella cheeses present a lower peak force compared to Cheddar, Gouda, and Provola. This indicates that mozzarella needs less force to start the formation of the filaments. Clear differences in filament strength are noticeable between Cheddar and Provola samples, with Provola showing higher filament strength. All six tested commercially available vegan mozzarella‐style cheeses showed no or very low filament formations, making them group very close in terms of filament strength. The peak force measured in vegan cheeses is lower than the peak force measured for Gouda, Provola, and Cheddar but equal to or superior to the peak force measured in mozzarella. This indicates that peak force alone is not enough to describe the stretchiness of cheese, and it needs to be evaluated together with other parameters. Large ranges of filament strength and peak force are shown for the four different presented Gouda cheeses. Different production processes and the aging period of each cheese can potentially be the driving forces of these differences. The breaking distance measured for all the samples is reported in Table 1. Nine out of fifteen of the dairy‐based cheeses showed values higher than the upper limit of the instrument (> 215 mm), while five out of six vegan cheeses showed values below the lower limit (*). The upper limit could be addressed by using a higher instrument capable of measuring values above 215 mm. By employing the proposed method at the optimized test conditions, it is possible to distinguish distinct stretch patterns among different types of cheeses, thereby enhancing the understanding of their textural differences.

### 3.5 Melting Test

As for stretchiness, meltability is also a significant functionality for certain cheeses. For this reason, not only the stretchiness but also the meltability were assessed on the same 21 cheese samples (cf. cheeses in Section 3.4), and the results are presented in Table 1. The meltability was calculated based on the percentage of the increase in the area after melting, as compared to the area before melting. This represents the expansion in the area due to melting. Among the tested samples, the six vegan cheeses generally exhibited no or low meltability compared to traditional dairy‐based cheeses (*p* < 0.05). An exception was observed for a dairy‐based Gouda cheese “Schärdinger Österreichischer Gouda,” which showed no significant difference than the vegan cheeses. This specific Gouda also demonstrated the lowest meltability among dairy‐based cheeses. This can be due to differences in the production and ripening process that may lead to a less flexible protein network responsible for low meltability.

These findings align with previous studies that indicated the absence of melting on starch‐based vegan cheeses, which represent most of the cheese analogues in the market. For instance, Grasso et al. (2024) found that various commercially available starch‐based cheese analogues, which contain a low proportion of proteins, did not exhibit melting upon heating. This suggests that the starch only provided integrity for its structure and did not induce melting upon heating. These findings are also in accordance with the results reported by Mattice and Marangoni (2020) on plant‐based cheeses made from corn starch and tapioca starch.

## 4 Conclusions

In this present study, an objective standardized method for measuring the stretch properties of melted cheeses using a texture analyzer is proposed. A new extensibility probe capable of analyzing a low amount of sample (i.e., down to 6.7 g) was also presented. A response surface methodology following a central composite design (CCD) was used to quantify the impact of the test parameters. This method was found to be useful in calculating optimized test parameters resulting in consistent stretch descriptors, namely stretch speed (i.e., 17.5 mm/s), stretch temperature (i.e., 66°C), and sample quantity (i.e., 6.7 g). Furthermore, the key stretch descriptors to describe the stretchiness of cheese were identified, including the force required to initiate stretching (i.e., peak force) and the filament strength as the force measured at a fixed distance of 40 mm. Total work done after a fixed distance (i.e., work at 40 mm) and breaking point were also considered, acknowledging the limit of the instrument (i.e., the max distance of 215 mm). The changes in mozzarella's stretch properties during storage can be followed using the proposed test parameters and stretch descriptors. Additionally, it enabled the clustering and differentiation of various cheeses based on their stretch properties. In this present study, both dairy‐based and vegan cheeses were analyzed. It is noted that hybrid cheeses (i.e., a mix between plant‐ and dairy‐based) gain popularity; thus, their stretch properties may be interesting to explore in further research. While this stretch measurement method is proposed as an essential tool for (vegan) cheese development, it is important to recognize its empirical nature. It is further noted that the results can be influenced by various factors, such as the probe geometry and the conditions used during the test.

## Author Contributions

**Pietro Andrigo:** conceptualization, formal analysis, investigation, methodology, software, visualization, writing – original draft. **Sara Spilimbergo:** supervision, writing – review and editing. **Belinda P. C. Dewi:** conceptualization, formal analysis, methodology, project administration, resources, supervision, visualization, writing – original draft, writing – review and editing.

## Ethics Statement

This study does not involve any human or animal testing.

## Conflicts of Interest

The authors declare no conflicts of interest.

## Supporting information


**Data S1.** Supporting Information.

## Data Availability

The data that support the findings of this study are available from the corresponding author upon reasonable request.
