# Supplementary material for: Development of a Novel Robust Approach for Unveiling the Stretchiness of Cheese
Source: J Texture Stud. 2025 Mar 4;56(2):e70012. doi: 10.1111/jtxs.70012 (PMC11876942; doi:10.1111/jtxs.70012)
Supplement: Supplementary file 1 — Data S1. Supporting Information. [file JTXS-56-e70012-s001.docx]

**Supplementary material 1**

Code to find the flattest point of the peak force equation (Python 3.10.12)

import numpy as np

from scipy.optimize import minimize

from scipy.optimize import approx_fprime

#Define the peakForce function

def peakForce(x):

    quantity, speed, temp = x

    return 118 - 7.38*quantity + 1.479*speed - 3.17*temp + 1.167*quantity**2 - 0.0280*speed**2

+ 0.0339*temp**2 + 0.1030*quantity*speed - 0.1512*quantity*temp - 0.0179*speed*temp

# Define the bounds for parameters

bounds = [(6, 10), (10, 20), (60, 70)]

#functions to normalize and denormalize parameters

def normalize(x, bounds):

    return [(x[i] - bounds[i][0]) / (bounds[i][1] - bounds[i][0]) for i in range(len(x))]

def denormalize(x, bounds):

    return [x[i] * (bounds[i][1] - bounds[i][0]) + bounds[i][0] for i in range(len(x))]

# Normalize initial guess (central point)

initial_guess = [8, 15, 65]

normalized_initial_guess = normalize(initial_guess, bounds)

# Compute the gradient of peakForce at point x

def antiFlatness(x):

    gradient = approx_fprime(x, peakForce, epsilon=1e-3)

    return np.sum(np.abs(gradient))

# Minimize the antiFlatness

result = minimize(lambda x: antiFlatness(denormalize(x, bounds)), normalized_initial_guess)

# Denormalize the solution

solution = denormalize(result.x, bounds)

#The flattest point of the peak force function is at the point:
#[6.688133783117797 g, 17.50720260747257 mm/s, 66.33778956258261 °C]
